# Books and Authors

**Published:** 1912-07

**Authors:** 


### BOOKS AND AUTHORS

**The Building Code and Tuberculosis Prevention.** 13-page pamphlet, by the **Committee on the Prevention of Tuberculosis of the C. O. S.** of the City of N. Y.

This is a most interesting discussion, although, naturally, applicable rather to problems of the metropolis and other large cities than to the smaller cities and villages of our especial territory.

There is one point in connection with this general subject that ought to be emphasized. When a building has been erected in good faith, in accordance with laws and accepted principles of architectural hygiene, that building should be left unmolested, barring minor changes in spite of revised laws, advance of sanitary science and architecture, and subject to ordinary conditions of supply and demand, to determine remodeling or adaptation to other than the primary use designed. To compel premature destruction or extensive rebuilding amounts to confiscation and, however desirable from the standpoint of philanthropy, will ultimately cause so serious a check upon investment and improvement of vacant property as to defeat the general object of building laws intended to safe-guard the health of occupants. If radical interference becomes necessary, the investor should be reimbursed, on exactly the same principal of ethics as governs compensation for condemned cattle, the condemnation of property for grade crossings, parks, play grounds, etc.

---

**Progressive Medicine,** Vol. 14, number 2, June 1, 1912. Edited by **Drs. H. A. Hare** and **L. F. Appleman,** Philadelphia; Lea & Febiger publishers. Quarterly $6 per year.

This number includes Hernia by Dr. Wm. B. Cooley; Surgery of the Abdomen exclusive of Hernia, by Dr. John C. A. Gerster; Gynecology by Dr. John G. Clark; Diseases of the Blood, Diathetic and Metabolic Diseases, etc., By Dr. Alfred Stengel, Ophthalmology by Dr. Edward Jackson. The general excellence of the series is maintained and the criticism occasionally made that reviewers write an original article expressing their own views and experience is not applicable here.

---

**Report of the Pathologic Department and the Department of Clinical Psychiatry, Central Indiana Hospital for Insane.** Vol. 4, 1909-10 and 1910-11, Indianapolis. 344 pages, illustrated.

Very little of this work is devoted to routine reports but every effort has been made to present concise statistics from which practical conclusions can be drawn. A good suggestion for reports in various fields of medicine may be derived from the way in which the necropsies are tabluated. For example, under such headings as acute mania, chronic mania, melancholia, etc., we find such statements as these: Acute Mania, 9 cases, males 5, females 4, (race and age statistics, duration of residence and cause of death for each) then, under each anatomic system, the incidence of each particular lesion is noted, with reference to detailed necropsy reports by number. Nervous system, scalp, congested, 1; cranial bones thin, 2; absence of diploe, 2; cerebrum congested, 1; the case numbers showing clearly, that the first and last abnormalities were associated in the same case and that the cranial bone abnormalities were associated in each of two other cases. This matter of association of abnormalities is very important and is often not indicated in reports or statistics are given in such a confusing way that, in attempting to compute percentages, we find that individual lesions add up to, say 131% of a suppositious total. Here we see at a glance that 2-3 of the cases of acute mania showed no nervous system lesions at all. It is just such impartial, clear-cut reporting of actual findings as are here given that will solve the problem of the pathologic basis of cerebral clinical diseases. To the Superintendent, Dr. George F. Edenharter and his able staff, are due the thanks of the profession both for the actual facts, positive and negative, bearing on record which may be readily adapted to reports in very different their views and experience, is not for establishing a form of this problem; and also for establishing a form of record which may be readily adapted to reports in very different fields of medicine.

---

**The Practical Medicine Series,** comprising ten volumes on the year's progress in medicine and surgery. The volumes are issued approximately monthly and each, dealing with a distinct field of medicine and surgery, is complete for the year previous to its publication. General Editors, **Gustavus P. Head** and **Charles L. Mix,** of Chicago; The Year Book Publishers, Chicago. Price of the series, $10.00 or each volume may be purchased separately. Vol. 1, **General Medicine,** edited by **Drs. Frank Billings** and **J. H. Salisbury** of Chicago. 404 pages, $1.50. Vol. 2, **General Surgery,** edited by Dr. **John B. Murphy,** Chicago, 616 pages, $2.00.

---

**Common Disorders and Diseases of Childhood. George Frederic Still,** M.A., M.D., F.R.C.P., London. Oxford University Press 1912. 813 pages, illustrated, $5.50.

As this is the second, revised edition of a work already familiar to the profession, a brief review is sufficient. Dr. Still emphasizes no particular part of the subject, introduces no novel features, makes no claim for the presentation of phenomenal cases. His book is sane, conservative, thorough, concise in its wording, reliable in its statements.

---

**Home Nurse's Handbook of Practical Nursing.** A manual for use use in home nursing classes, in Young Women's Christian Associations, in schools for girls and young women, and a working text-book for mothers, "practical" nurses, trained attendants, and all who have the responsibility of the home care of the sick. By **Charlotte A. Aikens,** author of "Hospital Management," "Hospital Training School Methods," "Primary Studies for Nurses," "Clinical Studies for Nurses." 12 mo. of 276 pages, illustrated. Philadelphia and London; W. B. Saunders Company, 1912. Cloth, $1.50 net.

The above clearly indicates the scope of the work and we need only add that the difficult task has been skillfully accomplished.

---

**Cyclopedia of American Medical Biography.** Cyclopedia of American Medical Biography. By **Howard A. Kelly,** M.D., Professor of Gynecologic Surgery at Johns Hopkins University, Baltimore. Two octavo volumes averaging 525 pages each, with portraits. Philadelphia and London; W. B. Saunders Company, 1912. Per set: cloth, $10.00 net; half Morocco, $13.00 net.

The period covered by this monumental work is 1610-1910. The author has wisely excluded living men, however famous. While obviously such a review is largely a work of compilation, Dr. Kelly has had the advantage of personal acquaintance with many of the men listed and in this way, has secured the knowledge necessary to a proper selection, from earlier biographic literature. Doubtless individuals will criticise the inclusion of some names, and deplore the omission of others but in no case can the author be accused of a personal bias. For example, we miss the name of Dr. Thomas Starr, the immigrant ancestor of many persons of that name—and of other surnames—in this country and Canada, who died in 1658, and also another name in which we have no personal interest—that of René Goupil, a brave young medical student who accompanied and shared the martyrdom of some of the Jesuit missionaries to Canada and New York. We are inclined to think that all of the pioneer physicians of what is now the United States and Canada should be included in such a work and feel confident that Dr. Kelly will welcome notes concerning them for use in a future edition.

